# Cetuximab inhibits colorectal cancer development through inactivating the Wnt/β-catenin pathway and modulating PLCB3 expression

**DOI:** 10.1038/s41598-024-59676-2

**Published:** 2024-05-09

**Authors:** Xiaohong Zhang, Wenming Zhou, Chenqu Wu, Jun Jiang, Qianqian Guo, Li Feng, Xun Cheng, Xingxing Zhang

**Affiliations:** 1 Fengxian District Center Hospital Graduate Student Training Base, Jinzhou Medical University, No. 6600 Nanfeng Road, Shanghai, 201499 China; 2 Endoscopy Center, Minhang Hospital, Fudan University, No. 170 Xinsong Road, Shanghai, 201199 China; 3 Department of Gastroenterology, Shanghai Jiaotong University Affiliated Sixth People Hospital South Campus, No. 6600 Nanfeng Road, Shanghai, 201499 China

**Keywords:** Cetuximab, Colorectal cancer, Wnt/β-catenin signaling pathway, PLCB3, Tumor progression, Cancer, Cell biology

## Abstract

Colorectal cancer (CRC) often necessitates cetuximab (an EGFR-targeting monoclonal antibody) for treatment. Despite its clinical utility, the specific operative mechanism of cetuximab remains elusive. This research investigated the influence of PLCB3, a potential CRC oncogene, on cetuximab treatment. We extracted differentially expressed genes from the GSE140973, the overlapping genes combined with 151 Wnt/β-Catenin signaling pathway-related genes were identified. Then, we conducted bioinformatics analysis to pinpoint the hub gene. Subsequently, we investigated the clinical expression characteristics of this hub gene, through cell experimental, scrutinized the impact of cetuximab and PLCB3 on CRC cellular progression. The study identified 26 overlapping genes. High expression of PLCB3, correlated with poorer prognosis. PLCB3 emerged as a significant oncogene associated with patient prognosis. In vitro tests revealed that cetuximab exerted a cytotoxic effect on CRC cells, with PLCB3 knockdown inhibiting CRC cell progression. Furthermore, cetuximab treatment led to a reduction in both β-catenin and PLCB3 expression, while simultaneously augmenting E-cadherin expression. These findings revealed PLCB3 promoted cetuximab inhibition on Wnt/β-catenin signaling. Finally, simultaneous application of cetuximab with a Wnt activator (IM12) and PLCB3 demonstrated inhibited CRC proliferation, migration, and invasion. The study emphasized the pivotal role of PLCB3 in CRC and its potential to enhance the efficacy of cetuximab treatment. Furthermore, cetuximab suppressed Wnt/β-catenin pathway to modulate PLCB3 expression, thus inhibiting colorectal cancer progression. This study offered fresh perspectives on cetuximab mechanism in CRC.

## Introduction

Colorectal cancer (CRC), an aggressive malignancy originating from the epithelial lining of the colon or rectum^1^, poses a significant public health concern globally^2^. Conventionally, CRC diagnoses rely on histopathological examinations^3^, with predominant clinical manifestations encapsulating alterations in bowel habits, abdominal discomfort, and rectal bleeding^4^. Notably, 20% of patients already have metastatic illness when they are diagnosed. Over the years, the therapeutic landscape for CRC has been revolutionized with the advent of molecular targeted therapies, including angiogenesis inhibitors and chemotherapeutic agents like oxaliplatin as well as fluorouracil^5^. Despite these therapeutic strides, around 17% of patients who receive curative resection continue to confront disease recurrence^6^. Thus, an enhanced understanding of the intricate molecular mechanisms propelling CRC progression and recurrence is pivotal in shaping innovative therapeutic strategies as well as boosting patient survival outcomes.

Established chemotherapy regimens for CRC, encompassing monoclonal antibodies and cytotoxic agents^7^, have solidified their efficacy within the clinical practice of CRC. Significantly, EGFR-targeting agents have demonstrated pronounced clinical activity^8^. The augmentation of standard combination chemotherapy with panitumumab and cetuximab, specifically in first-line therapy, has notably optimized clinical outcomes for patients grappling with metastatic CRC^9^. Cetuximab^10^, an IgG1 monoclonal antibody engineered to target EGFR, operates by impeding tyrosine kinase (TK) activity subsequent to EGFR binding^11^. This interaction orchestrates a disruption of intracellular signal transduction pathways, resulting in a myriad of outcomes: suppression of cancer cell proliferation, promotion of cell apoptosis, diminution in the production of matrix metalloproteinases^12^. The salient role of cetuximab in CRC treatment stems from its capacity to specifically target EGFR, thereby provoking various antitumor effects that can potentiate the efficacy of traditional chemotherapy^13,14^. Through the integration of cetuximab into treatment protocols, medical practitioners can provide metastatic CRC patients with a more individualized and precision-oriented therapeutic approach^13^. Such integration is anticipated to augment survival outcomes and mitigate treatment-related toxicity.

The Wnt/β-Catenin signaling cascade holds a vital function in mediating a plethora of cell processes, encapsulating differentiation, migration, and the maintenance of stem cell potency^15^. Numerous malignancies, including CRC, have been linked to the genesis and development of this pathway (dysregulation or abnormal activation). Within the context of CRC, genetic mutations or alterations in cardinal components of the Wnt/β-Catenin cascade, like APC, β-catenin (CTNNB1), and AXIN, would precipitate the unregulated activation^16^.

The study identified hub gene associated with cetuximab and the Wnt/β-Catenin pathway, and investigated the expression profile of PLCB3 in CRC as well as in vitro cellular assays. These insights enriched the understanding of cetuximab therapy and elucidated the role of PLCB3 in CRC.

## Material and methods

### Data acquisition and analysis

The Gene Expression Omnibus (GEO; https://www.ncbinlm.nih.gov/geo/)^17^ serves as a public repository housing high-throughput gene expression data. In the context of this study, we extracted the GSE140973 (http://www.ncbi.nlm.nih.gov/geo/query/acc.cgi) gene expression profile from the GEO database, encompassing a total of 114 samples. For our analysis, we selected 26 untreated (defined as the control group) and 29 cetuximab-treated CRC samples (defined as the case group), downloaded as a series matrix file. GEO2R^18^, an interactive web tool, was employed to facilitate the comparative analysis of these samples under specific conditions. We conducted an analysis of DEGs on the selected 55 samples from the GSE140973 dataset using GEO2R. This analysis incorporated fold change (FC) thresholds of > 1.3.The resulting data were generated by “ggplot2” package.

### Comprehensive analysis of overlapping genes

The Gene Set Enrichment Analysis (GSEA) database is a comprehensive resource for gene set analysis and functional annotation. For this study, we retrieved 151 genes implicated in Wnt/β-Catenin pathway from the GSEA database. The “VennDiagram” package in R language was employed to analyze overlapping genes between GSE140973-DEGs and the 151 pathway-related genes. To study the function of these overlapping genes in CRC, BP and KEGG analysis were conducted on the overlapping genes by “ClusterProfiler” package of R language.

### Effect of overlapping gene expression on survival probability of CRC patients

To assess the influence of overlapping genes on the survival of CRC patients, we utilized the Kaplan–Meier (KM) plotter, an online bioinformatics tool. This tool enabled us to construct survival curves for the 26 overlapping genes previously identified. This analysis was conducted to assess the effect of high and low gene levels on the post-progression survival (PPS) probability of CRC patients. The patient cohorts were stratified into high and low groups according to the average expression values of each gene. The log-rank test was employed to compute *P* values and hazard ratios (HRs).

### Expression analysis of PLCB3 in GSE140973 and TCGA-COAD databases

We first embarked on a comparative analysis of PLCB3 expression between the case and control groups within the GSE140973 gene expression dataset. We downloaded data from 455 colon adenocarcinoma (COAD) samples and the corresponding 41 non-tumor samples from the TCGA database (https://tcga-data.nci.nih.gov/tcga). Through the Clinical Biosignal House (https://www.aclbi.com/static/index.html#/tcga) for comprehensive analysis. We analyzed the levels of PLCB3 in these tumor samples, the obtained results were visualized using the “ggplot2” package in R programming language.

### Cell culture, treatment and transfection

CRC cell lines (Caco-2 and SW48), were purchased from the Chinese Academy of Sciences’ Culture Collection Center (Shanghai, China), they were propagated in DMEM enriched with 10% FBS under optimal conditions at 37 °C in a 5% CO_2_ humidified environment. For the treatment procedure, cells were subjected to 25 μg/ml of cetuximab, the Wnt inhibitor 21H7 (1 μM; Sigma-Aldrich, #SML0570, purity ≥ 98% (HPLC), USA), the Wnt activator IM12 (1 μM; Sigma-Aldrich, #SML0084, purity ≥ 98% (HPLC), USA)^19^, or specific combinations thereof, as denoted in the protocol. Caco-2 and SW48 cells were then transfected with either si-PLCB3-1 or si-PLCB3-2 by employing Lipofectamine 2000. Cells were removed for further examination 48 h after transfection.

### Cell proliferation assay

The Cell Counting Kit-8 (CCK-8) test^20^ was used to measure cell proliferation. Cells were seeded in 96-well plates. At predetermined time points of 24,48,72,96 hours, the CCK-8 reagent was supplemented, followed by the quantification of the OD at 450 nm utilizing a microplate reader. Proliferation curves were generated based on absorbance values to assess cell viability.

### Colony formation assay

The colony formation assay was conducted to assess the clonogenic potential of cells. Cells were seeded in 6-well plates and treated as indicated. After a period ranging from 10 to 14 days, crystal violet was employed to stain and manually count the colonies after they had been treated with methanol. The colony formation efficiency was computed as the percentage of seeded cells that successfully formed colonies, reflecting their ability to proliferate under the given conditions.

### Flow cytometry for cell cycle analysis

To remove RNA interference, cells were extracted, frozen in ice-cold 70% ethanol, and stained with propidium iodide (PI), which contains RNase A. Subsequent flow cytometry analysis facilitated the quantification of cells in the S, G0/G1, and G2/M phases.

### RNA extraction and quantitative real-time PCR (qRT-PCR)

TRIzol reagent was employed to extract total RNA from Caco-2 and SW48 cells in accordance with the manufacturer's instructions. Reverse transcription (RT) and quantitative PCR (qPCR) were performed using an Evo M-MLV RT Premix kit and SYBR Green Premix Pro Taq HS qPCR Kit (Accurate Biotechnology (Hunan) Co., Ltd). Following this, qRT-PCR was conducted employing gene-specific primers to assess the knockdown efficiency of PLCB3 in cells transfected with si-PLCB3-1 or si-PLCB3-2. The primer information used was as follows: PLCN3-forward: 5 ʹ-TATCTTCTTGGACCTGCTGACCGT-3ʹ and reverse: 5 ʹ-TGTGCCCTCATCTGTAGTTGGCTT-3ʹ; GAPDH-forward: 5ʹ- CGACCACTTTGTCAAGCTCA-3ʹ and reverse: 5ʹ-GGTTGAGCACAGGGTACTTTATT-3ʹ. The 2^−ΔΔCt^ technique was employed to compute relative gene expression, while GAPDH served as the internal reference.

### Western blotting (WB) assay

Caco-2 and SW48 cells, both treated with cetuximab and untreated controls, were lysed by RIPA buffer with protease inhibitors for protein extraction. The BCA assay was employed to detect protein content, and equivalent amounts of protein were segregated via SDS-PAGE. Subsequently, PVDF membranes received the transfer of proteins. Primary antibodies against PLCB3, β-catenin, and E-cadherin were then incubated for one night on the membranes at 4 °C after blocking by 5% non-fat milk. After being washed, the membranes were treated with the proper secondary antibodies that were HRP-conjugated. An improved chemiluminescence detection technique was employed to see the protein bands, and ImageJ was applied to analyze the densitometry data and calculate the protein expression.

### Transwell assay

Migration and invasion assays were performed using Transwell chambers or Transwell chambers pre-coated with Matrigel according to the manufacturer's (BD Biosciences, Bedford, MA, USA) protocol. Briefly, for migration and invasion assays, 5 × 104 cells were cultured in 200 µl serum-free medium in the upper chamber of an inserted 24-well plate, and then 600 µl of medium containing 10% FBS was added to the lower chamber. After 24 h and 48 h of incubation at 37 °C for migration and invasion experiments, respectively, non-migrated or non-invaded cells were gently removed, and the invaded cells in the lower filter were fixed with 4% polymethanol for 20 min, stained with 0.1% DAPI and counted under a microscope. Experiments were performed in triplicate.

#### Dual-luciferase reporter assay

Genomeditech (Shanghai, China) donated the TCF/LEF1-Luc reporter plasmid. In order to check the effectiveness of the transfection process, cells were also co-transfected with the Renilla luciferase vector and the PCLB3 promoter-luciferase reporter construct. After treatments, which included exposure to DMSO as a solvent control for certain treatments, cells were lysed. To account for variability in transfection effectiveness between samples, firefly luciferase activity was standardized to detect Renilla luciferase activity.

#### Statistical analysis

All experiments were independently repeated three times or specified at figure legends. Data were showed as mean ± standard deviation (SD). The student’s t-test (two-tailed) or the analysis of one-way ANOVAs was employed to examined the differences. All statistical analysis was performed by GraphPad Prism 6.0 (GraphPad Software Inc., San Diego, CA, USA). A value of *P* < 0.05 was recognized as significant.

## Results

### Identification and functional analysis of GSE140973-DEGs related to Wnt/β-Catenin signaling pathway

**Figure 1:**
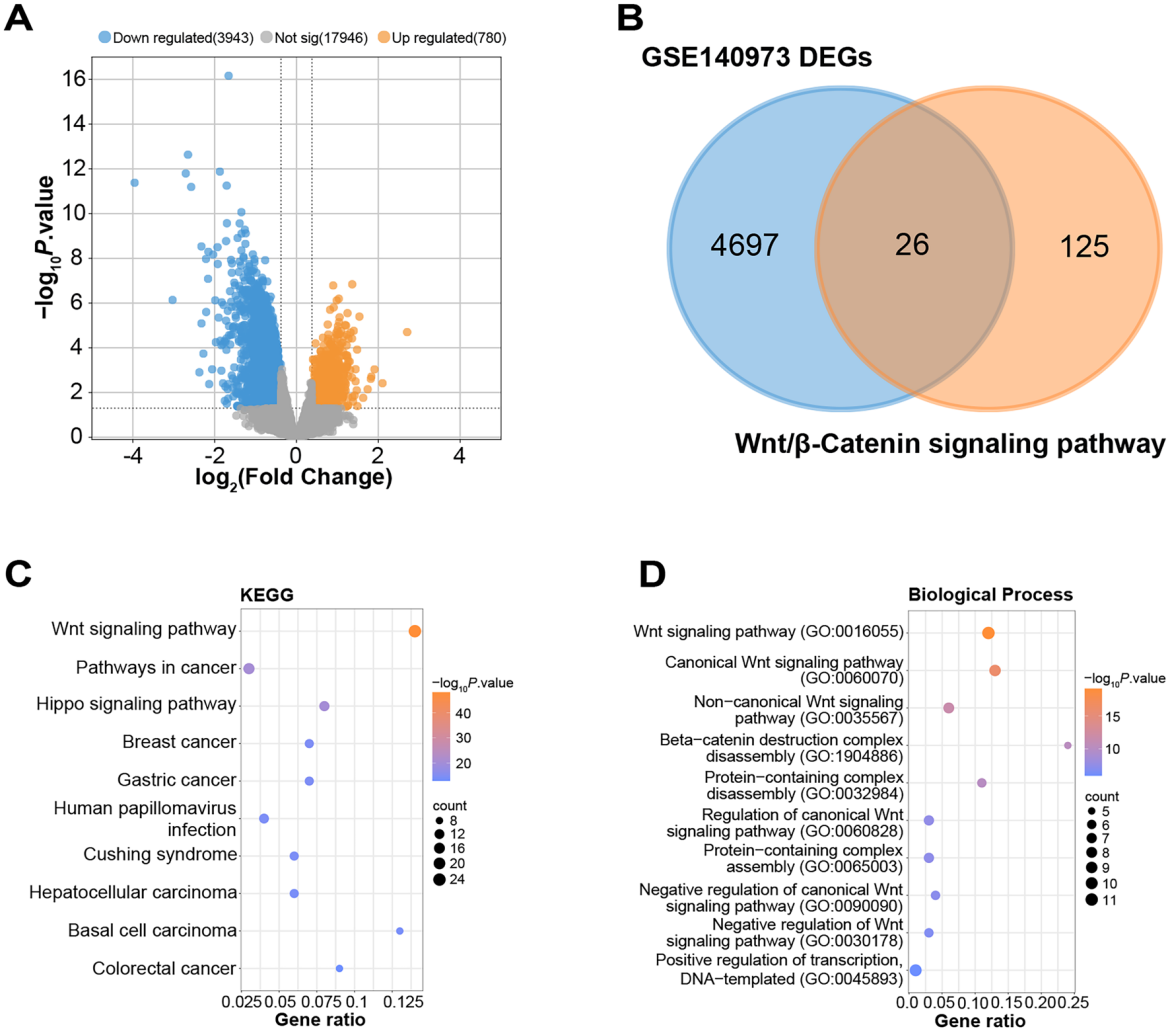
Identification and characterization of DEGs associated with the Wnt/β-Catenin signaling pathway. (**A**) Volcano plot, the DEGs between case and control samples in the GSE140973 dataset, identifying 780 up-regulated and 3943 down-regulated genes. The vertical lines correspond to 2.0-fold up and down, respectively, and the horizontal line represents a *P* value of 0.05. The orange points in the plot represent up-regulated DEGs, and the blue points represent down-regulated DEGs. (**B**) Venn diagram displaying the overlap of 26 genes between the 4723 DEGs and the 151 genes related to the Wnt/β-Catenin signaling pathway. (**C**) Bubble plots showing the top 10 significantly enriched in the KEGG pathways^42–44^. The size of the bubbles corresponds to the ratio of genes associated with that term or pathway. (**D**) Bubble plots showing the top 10 significantly enriched in the BP categories. The size of the bubbles corresponds to the ratio of genes associated with that term or pathway.

GEO2R tool was employed to analyze the DEGs between case and control samples in the GSE140973 dataset. A total of 780 DEGs exhibited upregulation, while 3943 DEGs demonstrated downregulation, as depicted in Fig. 1A. Subsequently, the “VennDiagram” package recognized 26 overlapping genes from the 4723 DEGs and 151 Wnt/β-Catenin signaling pathway-related genes (Fig. 1B). Next, the top 10 enriched KEGG pathways for these DEGs contained Hippo signaling pathway, and Cushing syndrome, among others (Fig. 1C). In addition, BP functional analysis of DEGs revealed enrichment of items included Wnt signaling pathway, kinase binding, intracellular membrane-bounded organelle, and nucleus (Fig. 1D).

### PLCB3 emerges as the central hub gene

**Figure 2:**
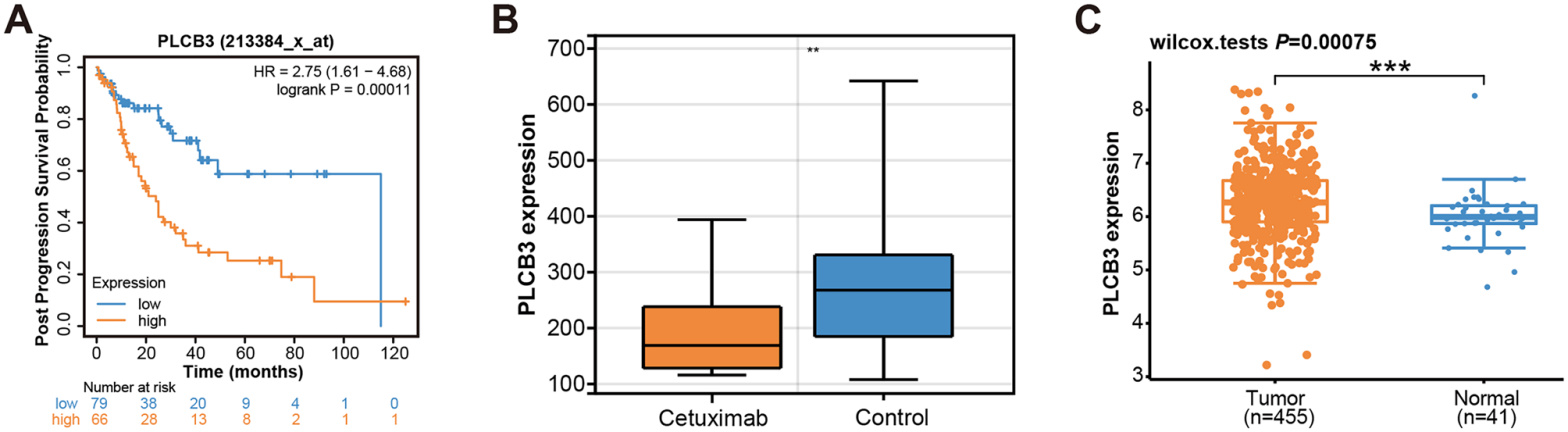
KM survival analysis of PLCB3 gene in CRC patients with expression analysis in different datasets. (**A**) Survival curves of PPS probabilities for PLCB3. The horizontal axis represents survival time in years, while the vertical axis represents the probability of PPS. Orange lines correspond to high gene expression, while blue lines indicate low gene expression. HR and *P* values for high and low expression for each gene are shown in the upper right corner of each plot. (**B**) Differential expression of PLCB3 between case and control groups in the GSE140973 gene expression dataset. (**C**) The Wilcoxon rank sum test compared PLCB3 expression levels between 455 COAD samples and 41 adjacent non-tumor samples from the TCGA database. *P < 0.05, **P < 0.01, ***P < 0.001.

We analyzed the influence of high/low expression of the 26 overlapping genes on the PPS probability of CRC patients using the KM plotter website, and obtained significant results (Fig. 2A). High expression of the remaining genes PLCB3 resulted in lower PPS probability and shorter survival time. This finding underscored the profound influence of differential gene expression on the prognosis of CRC patients. Among these genes, PLCB3 stands out as particularly significant, demonstrating the smallest *P* value (0.00011) based on the log-rank test. With a hazard ratio (HR) of 2.75 and a confidence interval (CI) ranging from 1.61 to 4.68, PLCB3 exerts the most pronounced impact on the outcomes of colorectal cancer (CRC) patients. Consequently, PLCB3 was regarded as a hub gene for in-depth analysis in this study. In the subsequent expression analysis, we found that PLCB3 exhibited low expression in the case group of the GSE140973 gene expression profile (Fig. 2B), indicating that PLCB3 level might be influenced by cetuximab treatment. Concurrently, our analysis of the TCGA database demonstrated elevated PLCB3 expression levels within COAD tumor tissues, suggesting that this gene may function as an oncogene (Fig. 2C).

### Toxic effects of cetuximab therapy on CRC cells

**Figure 3:**
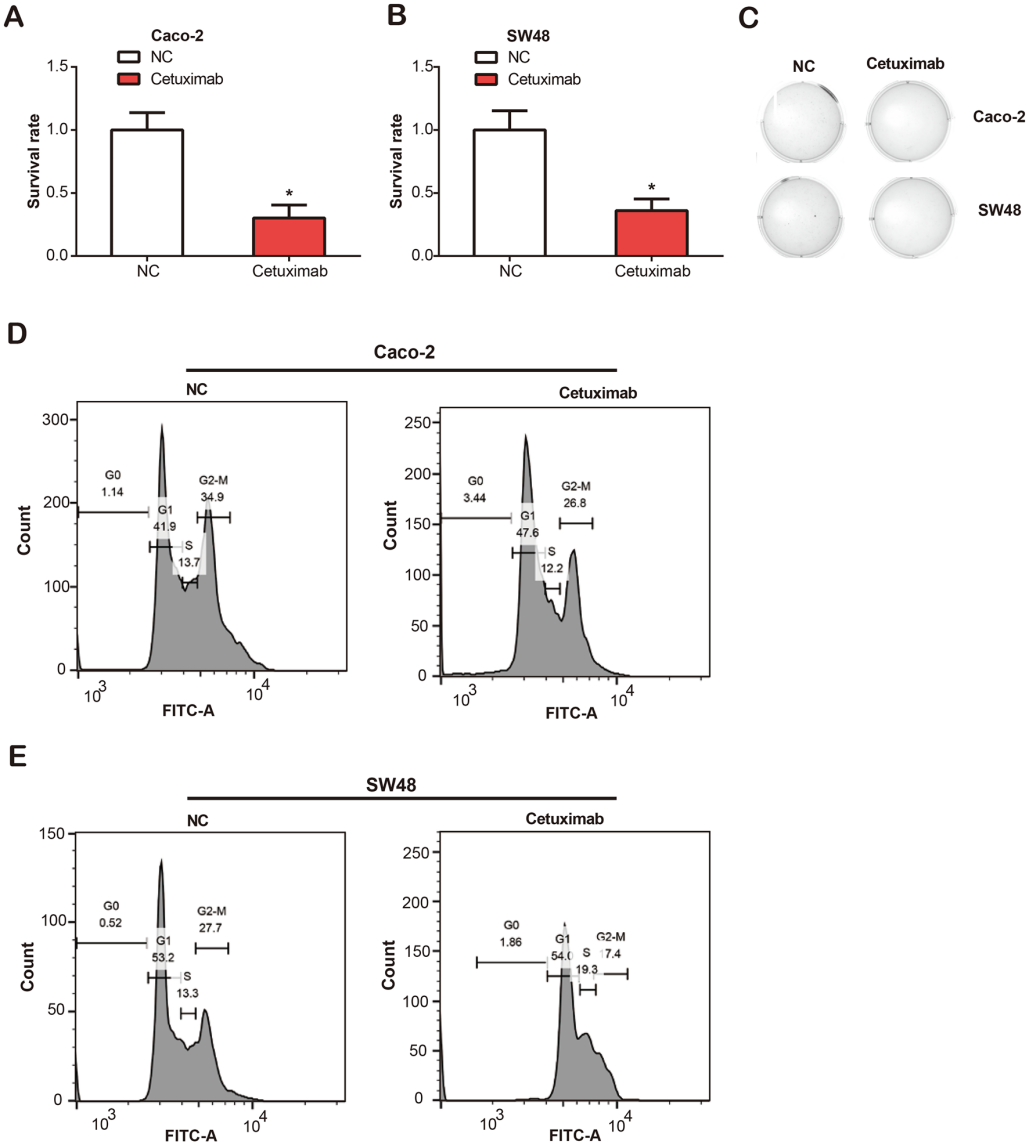
Impact of cetuximab treatment on proliferation, viability, and cell cycle progression of CRC cell lines. (**A**, **B**) CCK-8 technique was used to detect the effect of cetuximab on the proliferation of two colorectal cancer cell lines (Caco-2 and SW48). *P < 0.05. (**C**) Colony formation assay Caco-2 and SW48 cell viability after treatment with 21H7 or cetuximab. (**D**, **E**) The effect of 21H7 or cetuximab treatment on the cell cycle progression of Caco-2 and SW48 cells was examined by flow cytometry.

To measure the effect of cetuximab treatment on CRC cell lines, we initially conducted a CCK-8 assay. The survival rate of CRC cells with cetuximab at a concentration of 25 μg/ml was saliently reduced (Fig. 3A,B). Subsequently, we carried out colony formation assays, and a reduction was observed in the viability of both Caco-2 and SW48 cells post-cetuximab treatment (Fig. 3C). Concurrently, flow cytometric cell cycle analysis demonstrated an augmented proportion of Caco-2 and SW48 cells residing in the G0/G1 phase post-cetuximab treatment (Fig. 3D,E). Taken together, these results underscore the efficacy of cetuximab in attenuating cell growth and inducing G0/G1 phase cell cycle arrest in CRC cell lines, emphasizing its potential as a treatment target.

### PLCB3 knockdown inhibits cell proliferation, invasion and migration in CRC

**Figure 4:**
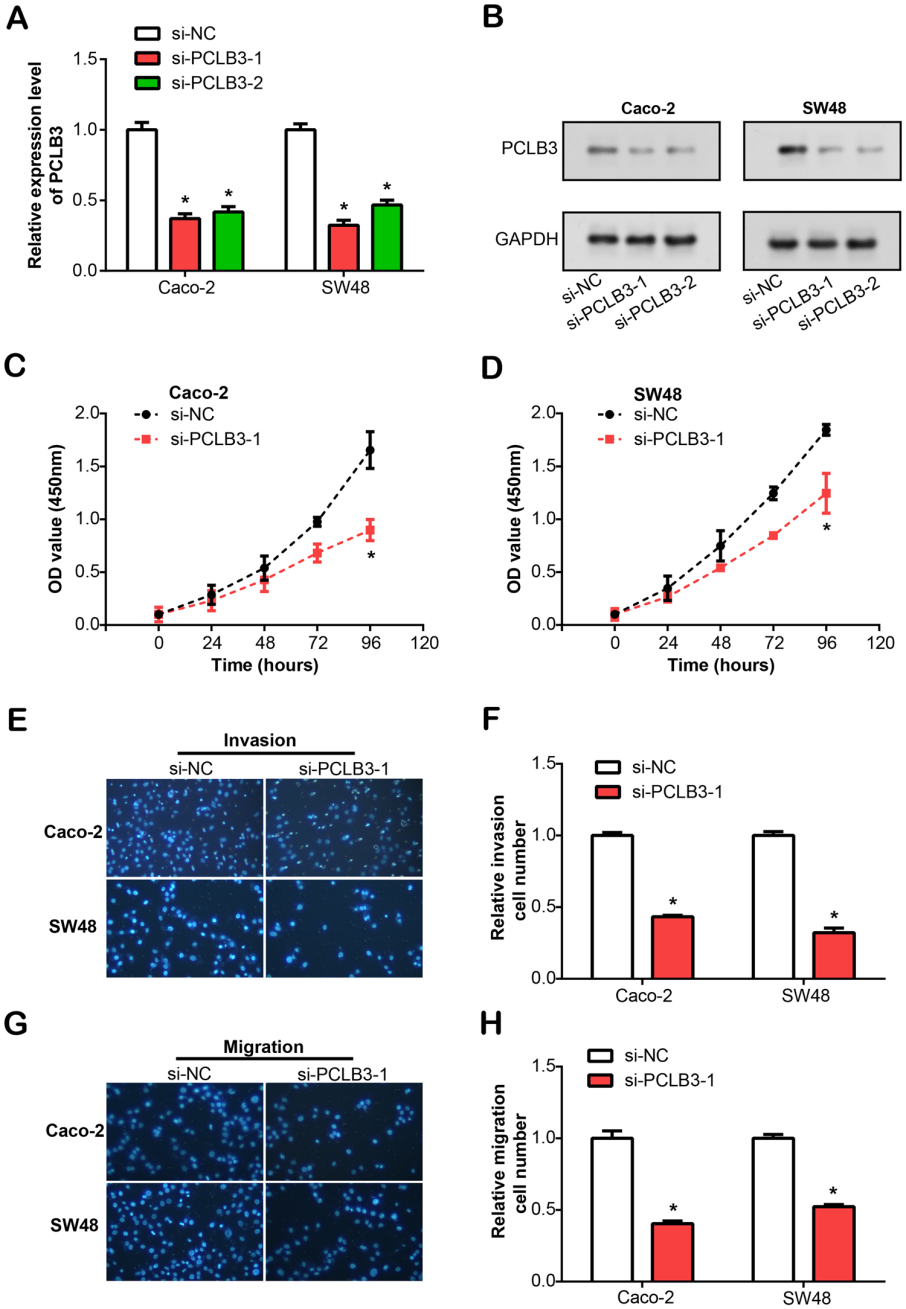
The PLCB3 knockdown impact on proliferation, migration, and invasion of CRC cell lines. (**A**) The knockdown efficiency of PLCB3 siRNA (si-PLCB3-1, si-PLCB3-2) in Caco-2 and SW48 cells was detected by qRT-PCR. (**B**) The knockdown efficiency of PLCB3 siRNA (si-PLCB3-1, si-PLCB3-2) in Caco-2 and SW48 cells was verified by WB. Gels/blots were cropped from different regions of the same gel and are delineated by clear dividing lines. (**C**, **D**) CCK-8 detection growth curve results of cell proliferation after knockdown of PCLB3 (si-PLCB3-1) in Caco-2 and SW48 cells. (**E**, **F**) Transwell detected the effect of PLCB3 knockdown (si-PLCB3-1) on the invasion of Caco-2 and SW48 cells. (**G**, **H**) Transwell detected the effect of PLCB3 knockdown (si-PLCB3-1) on the migration of Caco-2 and SW48 cells. *P < 0.05.

In subsequent experiments, we transfected PLCB3 small interfering RNAs (si-PLCB3-1, si-PLCB3-2) into Caco-2 and SW48 cells. The efficacy of transfection was confirmed through qRT-PCR analysis, as shown in Fig. 4A. Additionally, WB analysis was performed to further validate the knockdown of PLCB3 expression in both cell lines (Fig. 4B). Indicated the impact of si-PLCB3-1 and si-PLCB3-2 on the protein levels of PLCB3 in Caco-2 and SW48 cells. These findings collectively demonstrated the successful modulation of PLCB3 expression at both the mRNA and protein levels in the experimental cell lines. Subsequently, the growth curve results from the CCK-8 assay demonstrated PLCB3 knockdown suppressed the growth of both Caco-2 and SW48 cells (Fig. 4C,D). Furthermore, results from the Transwell assay indicated a reduced trend in the migrating and invading capacity of both cells with PLCB3 knockdown, when compared with the control group (Fig. 4E–H). Overall, our findings demonstrate PLCB3 acts a crucial role in regulating CRC cell activities.

### Cetuximab suppresses PLCB3 expression via inhibition of the Wnt/β-Catenin signaling pathway

**Figure 5:**
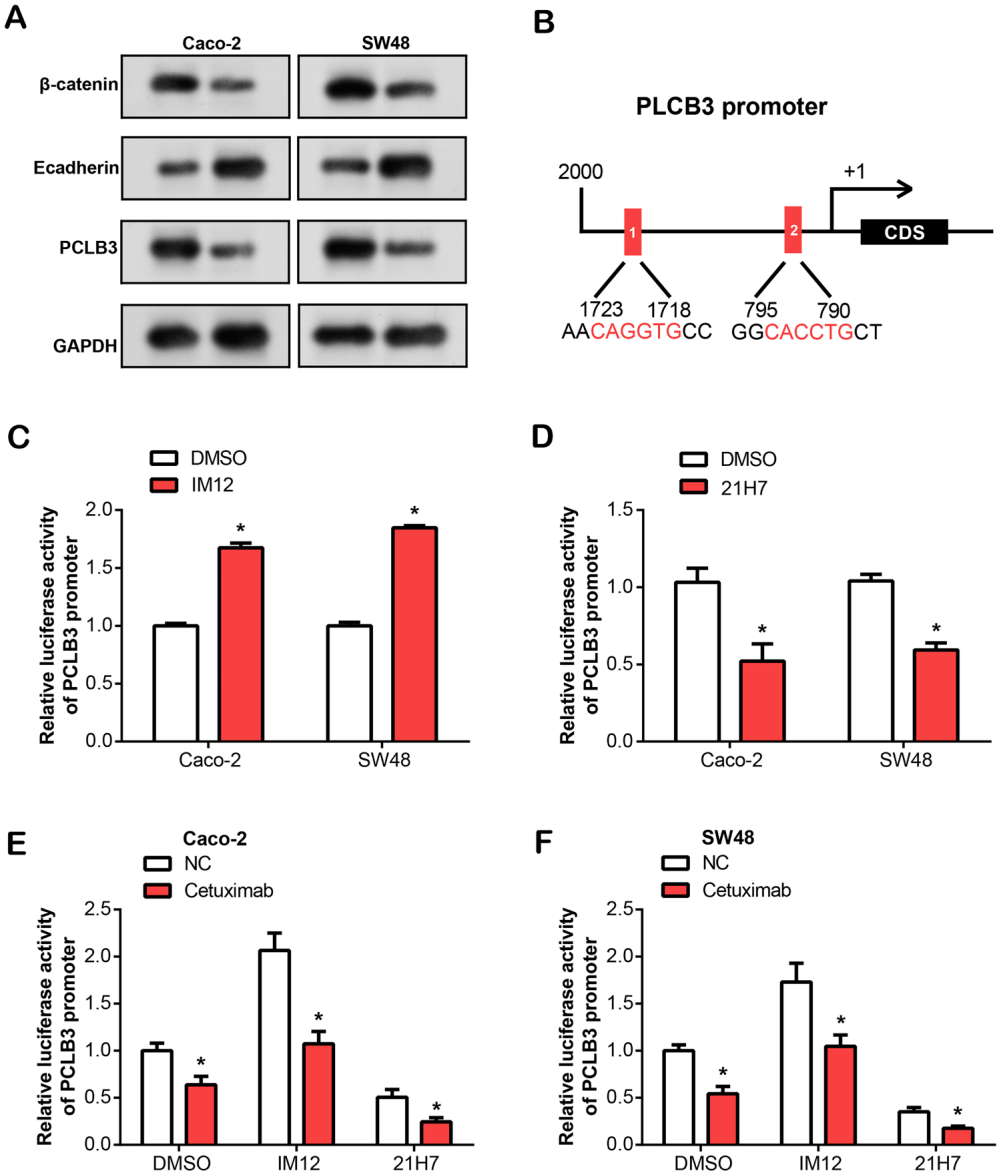
Cetuximab treatment alters protein expression and affects PLCB3 promoter activity associated with Wnt/β-catenin signaling in CRC cells. (**A**) The protein expression levels of β-catenin, Ecadherin and PCLB in Caco-2 and SW48 cells were detected by WB after cetuximab treatment. Different gels were used, and blots were combined for presentation, with clear delineations between them. (**B**) Schematic representation of two predicted TCF/LEF binding sites in the PLCB3 promoter region. (**C**–**F**) Dual-luciferase assays to assess PLCB3 promoter activity in Caco-2 and SW48 cells under various conditions: (**C**) Wnt inhibitor (21H7) treatment, (**D**) Wnt activator (IM12) treatment, (**E**, **F**) Cetuximab combined with 21H7 or IM12 treatment. *P < 0.05.

WB analyses demonstrated significant alterations in protein expressions following cetuximab treatment. Specifically, the protein expressions of β-catenin and PLCB3 reduced, whereas the expression of E-cadherin (a protein downstream of β-catenin)^21^ protein increased (Fig. 5A). The observed decrease in β-catenin levels suggests inhibition of its signaling cascade, which may contribute to the therapeutic effect of cetuximab. In addition, the downregulation of PLCB3 suggests that intracellular signaling pathways are regulated, highlighting the complex mechanisms by which cetuximab affects cellular processes. Moreover, in the promoter region of PLCB3, we discovered two possible TCF/LEF effector elements^22^ with the consensus sequence CAGGTG (E-box motif). Analysis of the PLCB3 promoter region revealed two significant binding sites located upstream of the coding sequence (CDS). The first site (1) is positioned at − 1723 to − 1718 relative to the transcription start site, containing the sequence AACAGGTGCC. The second site (2) is located closer to the CDS at positions − 795 to − 790, with the sequence GGCACTGCT. Both sites are indicated by red boxes, suggesting potential regulatory roles in gene expression (Fig. 5B). Previous research has shown that IM12, an activator of the same system, greatly raises the levels of β-catenin, whereas 21H7 is a specific inhibitor of the Wnt/β-catenin pathway, capable of destroying β-catenin. Therefore, we assessed the activity of the PLCB3 promoter in CRC cells under various treatments using a dual-luciferase assay. As shown in Fig. 5C–F, the activity of the PLCB3 promoter was increased in CRC cell lines treated with the Wnt pathway activator IM12. Instead, it decreased in patients treated with the Wnt pathway inhibitor 21H7. However, attenuation of PLCB3 promoter activity was observed in cells with cetuximab and IM12 or 21H7.

### Cetuximab inactivates Wnt/β-catenin pathway to regulate PCLB3 expression and inhibit colorectal cancer progression

**Figure 6:**
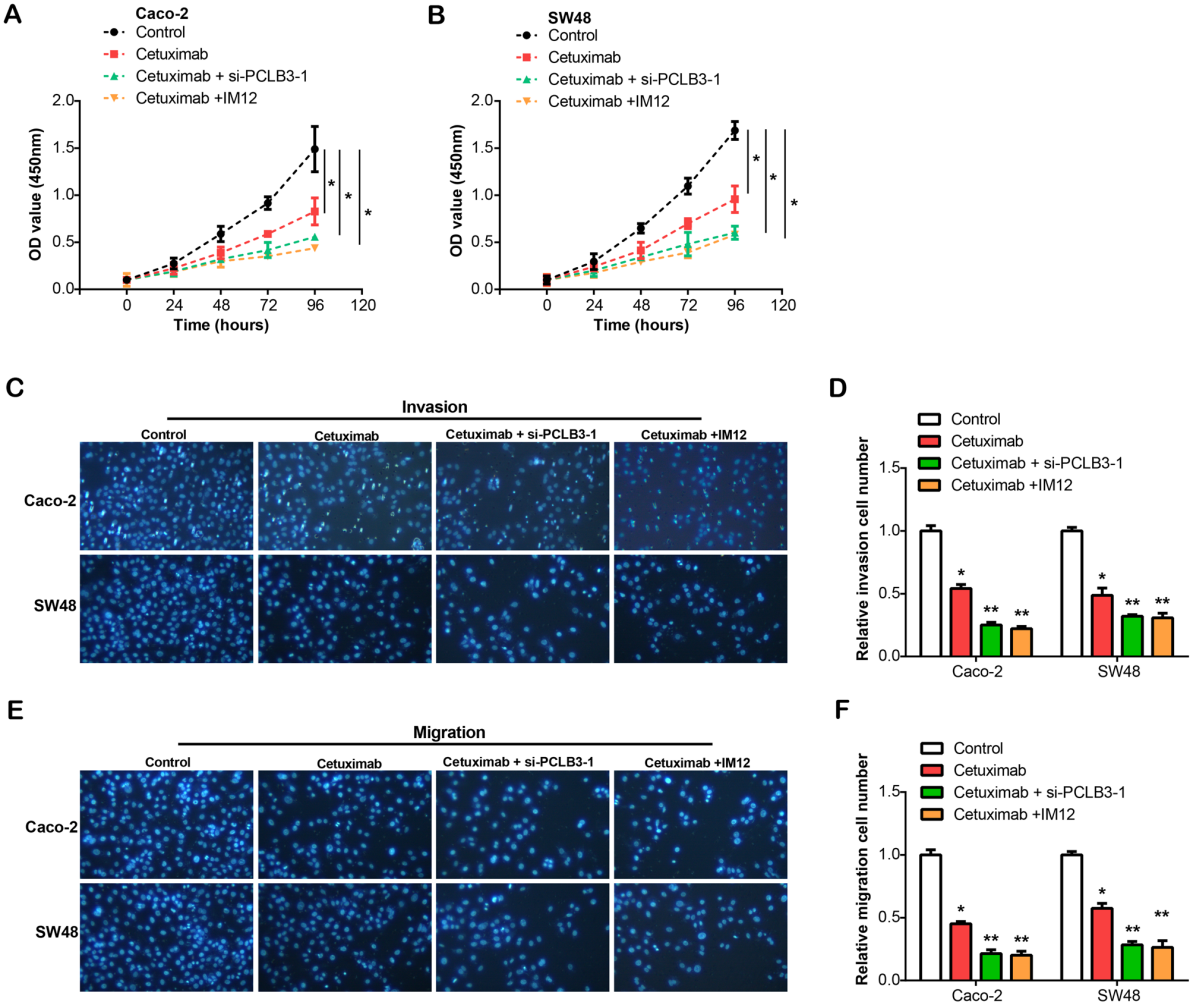
Effects of cetuximab, si-PCLB3, and IM12 on proliferation, migration, and invasion of CRC cells. (**A**, **B**) CCK-8 assay for the proliferation of CRC cells (Caco-2 and SW48) under different conditions: Control, Cetuximab treatment, Cetuximab combined with si-PCLB3-1, and Cetuximab combined with IM12. (**C**, **F**) Transwell assay for migration and invasion of CRC cells (Caco-2 and SW48) under different conditions: control, cetuximab treatment, cetuximab combined with si-PCLB3, and cetuximab combined with IM12. *P < 0.05, **P < 0.01.

We examined the proliferative abilities of colorectal cancer cells (Caco-2 and SW48) using a CCK-8 assay (Fig. 6A,B). Notably, relative to the control group, the proliferative capacity of CRC cells under cetuximab treatment was considerably diminished. This inhibition of cell proliferation was further amplified when cetuximab was administered in conjunction with si-PLCB3-1 or a Wnt pathway activator (IM12). Analogously, our Transwell assay indicated a significant reduction in the migrating and invading abilities of CRC cells for all three treatment groups compared to the control. Remarkably, the decline in cell migration and invasion was further accentuated upon the addition of either si-PLCB3-1 or IM12 to the cetuximab treatment (Fig. 6C–F). The combined treatments of cetuximab, si-PLCB3-1, and Wnt activator significantly inhibit CRC cell progression, highlighting a promising therapeutic strategy for CRC treatment.

## Discussion

CRC, with its high morbidity and mortality rates worldwide, has emerged as a significant global public health concern. It is a common malignant tumor of the digestive system, and its treatment options are continuously evolving. Currently, a variety of treatment modalities, including endoscopy and surgical resection, radiotherapy, immunotherapy, palliative chemotherapy, targeted therapy, as well as extensive surgery and local ablation for metastatic lesions, are widely employed. These treatments effectively inhibit the progression of cancer and extend overall survival^23^. Cetuximab has shown promise in various cancer studies^24^. For instance, a study by Lucas Maahs et al. explored the use of cetuximab in preventing anemia in head and neck cancer patients undergoing radiotherapy^25^. This research highlights the multifaceted applications of cetuximab beyond its direct anti-tumor effects. Globally, a considerable number of patients have experienced improved outcomes with cetuximab treatment, further underscoring its therapeutic potential. Besides, cetuximab has been reported to enhance RSL3-induced ferroptosis by inhibiting the Nrf2/HO-1 axis^26^. This process is facilitated by p38 MAPK activation, a critical player in cell signaling pathways associated with cell survival and apoptosis. This evidence implies that cetuximab's beneficial effects might extend beyond its direct action on EGFR, possibly involving intricate interactions with various cellular pathways, including those governing ferroptosis^27^. As our understanding of cetuximab's mechanisms of action expands, it is hoped that its therapeutic efficacy can be further optimized, leading to improved outcomes in CRC.

The importance of Wnt/β-Catenin pathway in the development and treatment of cancer has long been acknowledged^28^. Colorectal cancer exhibits evidence of activation in the Wnt signaling pathway, which is associated with the loss of function of the tumor regulatory factor APC. Wnt activation has been observed in breast cancer, lung cancer, and hematologic malignancies, contributing to tumor recurrence. The cross-talk between the Wnt pathway and the Notch and Sonic Hedgehog pathways holds significant implications for therapeutic interventions in cancer^29^. It is essential for cellular functions like migration, survival, differentiation, and proliferation, all of which are dysregulated in cancer. Consequently, this pathway represents an attractive therapeutic strategy in oncology. Studies suggested the therapeutic potential of cetuximab in combination with β-caryophyllene in treating KRAS-mutated CRC^30^. Inspired by these findings, we queried the GSEA database for genes associated with Wnt/β-Catenin pathway, retrieving 151 related genes. We subsequently identified 26 overlapping genes from these 151 genes and the DEGs from the GSE140973 dataset. The enrichment analysis of these overlapping genes in GO and KEGG revealed significant associations with processes and pathways such as Wnt signaling pathway^31^, and kinase binding. Additionally, connections were found with the Hippo signaling pathway^32,33^ and Cushing syndrome, among others. These results indicate a potential interplay between these overlapping genes and the aforementioned pathways and processes, implying their substantial roles in the pathogenesis and progression of CRC. It also suggests that these genes may be potential therapeutic targets for CRC, warranting further investigation.

Following the identification of overlapping genes, we further examined the prognostic implications using KM survival curve. Notably, we found that the high expression of PLCB3 was associated with a poorer prognosis. Strikingly, PLCB3 showed the most significant prognostic impact. PLCB3, short for Phospholipase C Beta 3, belongs to the phosphoinositide-specific phospholipase C family^34,35^. These enzymes are pivotal for intracellular signal transduction, playing a vital function in processes like cell growth, differentiation, and apoptosis. Emerging evidence has implicated aberrations in PLCB3 expression in various cancers^36–38^, indicating its potential role as oncogene or suppressor gene. The research finding indicated that PLCB3 has been identified through RNA sequencing in inflammation and myocardium as a crucial gene involved in the promotion of M2 macrophage polarization by UMSC-Exo. By increasing the expression of miR-24-3p in macrophages, the expression of PLCB3 and activation of the NF-κB pathway in the inflammatory environment were effectively suppressed, leading to a significant enhancement of M3 macrophage polarization^39^. Using bioinformatics methods, we screened for differentially expressed glycolysis genes between HNSCC tissues and normal head and neck tissues from the TCGA database. In the validation cohort, we identified a set of six glycolysis genes significantly associated with overall survival (OS). Further analysis revealed that one of them, PLCB3, may serve as an independent risk factor for HNSCC prognosis, demonstrating its potential in predicting patient survival^40^. Our study demonstrated that PLCB3 was downregulated in CRC samples treated with cetuximab (GSE140973-case group), while it was upregulated in the TCGA-COAD samples. This observation suggests a regulatory role of cetuximab on PLCB3 expression, potentially underlying its therapeutic efficacy.

Our in vitro investigations revealed that cetuximab exhibited potent anti-proliferative effects on CRC cell lines, resulting in significant cell cycle arrest at the G0/G1 phase. Furthermore, PLCB3 knockdown demonstrated a similar inhibitory influence on cell proliferation, migration, and invasion, thereby underscoring the crucial role PLCB3 might play in CRC pathogenesis. Interestingly, our study also uncovered a potential regulatory mechanism by which cetuximab operates. Our analyses suggested that cetuximab treatment culminates in the downregulation of both β-catenin and PLCB3, critical components of the Wnt/β-catenin pathway, and concurrently elevates E-cadherin expression. This observation was further supported by our findings from the dual-luciferase assay, which demonstrated the potential of cetuximab to modulate PLCB3 promoter activity through Wnt/β-catenin pathway. The results indicate a possible interplay between cetuximab, PLCB3, and Wnt/β-catenin pathway, shedding light on the mechanistic basis of cetuximab therapeutic efficacy. Lastly, our data demonstrated that the combination therapy involving cetuximab, si-PLCB3, and a Wnt activator (IM12) contributed to a pronounced down-regulation in CRC cell proliferation, migration, and invasion^41^. This therapeutic strategy may harness the anti-tumor properties of these agents, thus paving the way for improved CRC treatment modalities. Together, the results provide a promising perspective into PLCB3 and cetuximab in CRC, and underscore the potential of targeted therapy against PLCB3 and Wnt/β-catenin pathway in enhancing the efficacy of cetuximab-based CRC treatment.

## Conclusion

In conclusion, our comprehensive study aimed to elucidate the roles of cetuximab and the Wnt/β-catenin pathway in colorectal cancer (CRC). Through our study, we succeeded in identifying overlapping genes associated with GSE140973-DEGs and the Wnt/β-catenin pathway, and determined the critical role of the hub gene PLCB3 in CRC. Importantly, our findings emphasized the critical role of cetuximab in inhibiting the Wnt/β-catenin pathway, thereby modulating PLCB3 expression and effectively inhibiting CRC progression. We emphasized that our study not only contributes to a deeper understanding of the molecular mechanisms of CRC, but also revealed the specific effects of cetuximab on the Wnt/β-catenin pathway. The identification of PLCB3 as a key mediator further deepened our understanding of the intricate signaling network involved in CRC. These insights provided a foundation for more effective targeted therapeutic strategies in the ongoing fight against colorectal cancer and offered a promising avenue for improving outcomes for CRC patients.

### Supplementary Information

Supplementary Information 1.

Supplementary Information 2.

Supplementary Information 3.

Supplementary Information 4.

Supplementary Information 5.

Supplementary Information 6.

Supplementary Information 7.

Supplementary Information 8.

Supplementary Information 9.

Supplementary Information 10.

Supplementary Information 11.

Supplementary Information 12.

## Data Availability

GEO database: The GSE140973 gene expression profile can be accessed at (https://www.ncbi.nlm.nih.gov/geo/query/acc.cgi) with the accession number: GSE140973. TCGA database: Data related to COAD samples were extracted from (https://tcga-data.nci.nih.gov/tcga) and Clinical Biosignal House (https://www.aclbi.com/ static/index.html#/tcga). Other datasets: The datasets used and/or analyzed during the current study are available from the corresponding author on reasonable request.
