# Supplementary figures and images for: Cetuximab inhibits colorectal cancer development through inactivating the Wnt/β-catenin pathway and modulating PLCB3 expression
Source: Sci Rep. 2024 May 9;14:10642. doi: 10.1038/s41598-024-59676-2 (PMC11081956; doi:10.1038/s41598-024-59676-2)

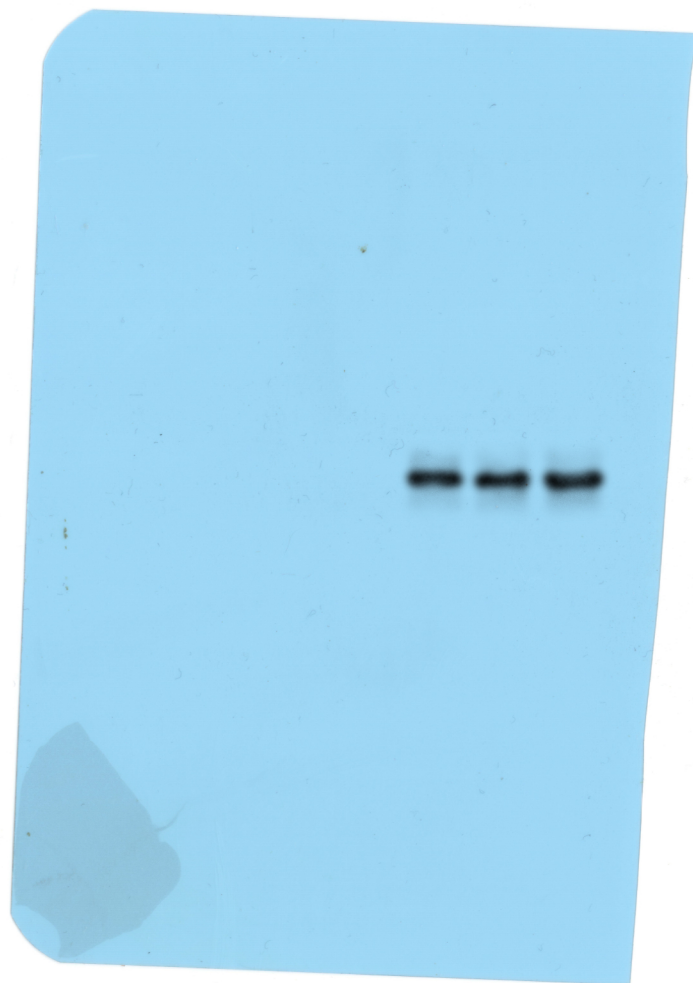

Supplement: Supplementary file 1 — Supplementary Information 1. [file 41598_2024_59676_MOESM1_ESM.pdf]

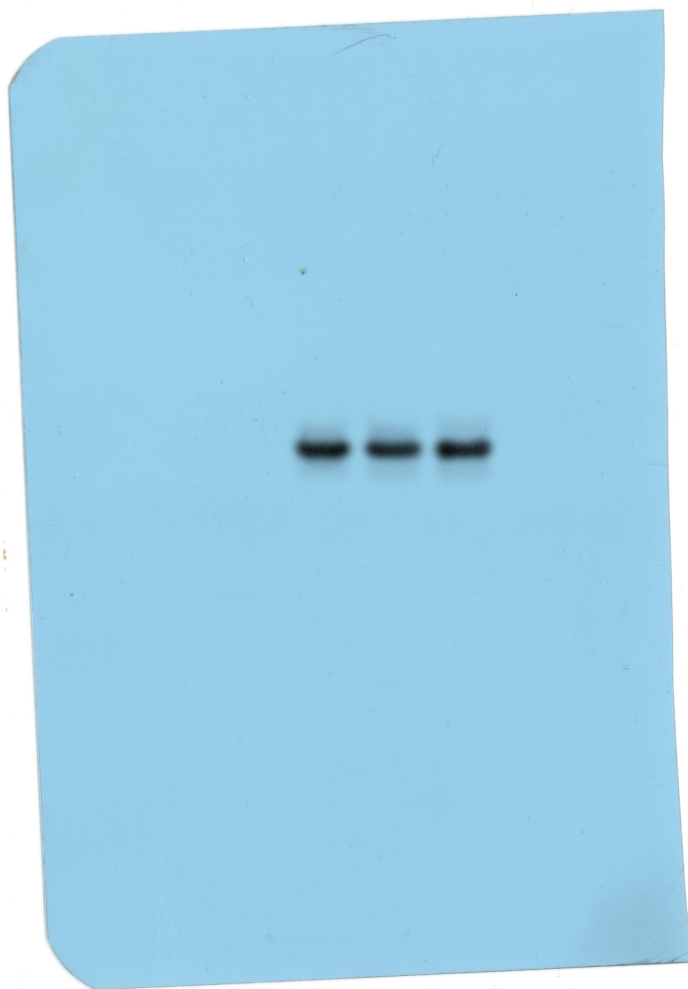

Supplement: Supplementary file 2 — Supplementary Information 2. [file 41598_2024_59676_MOESM2_ESM.pdf]

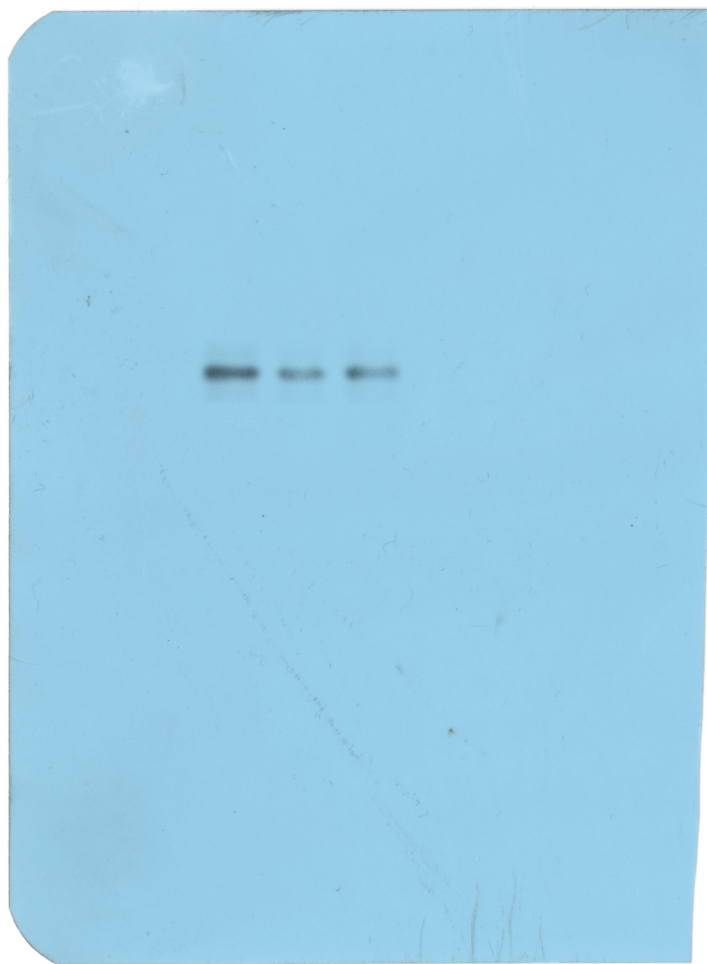

Supplement: Supplementary file 3 — Supplementary Information 3. [file 41598_2024_59676_MOESM3_ESM.pdf]

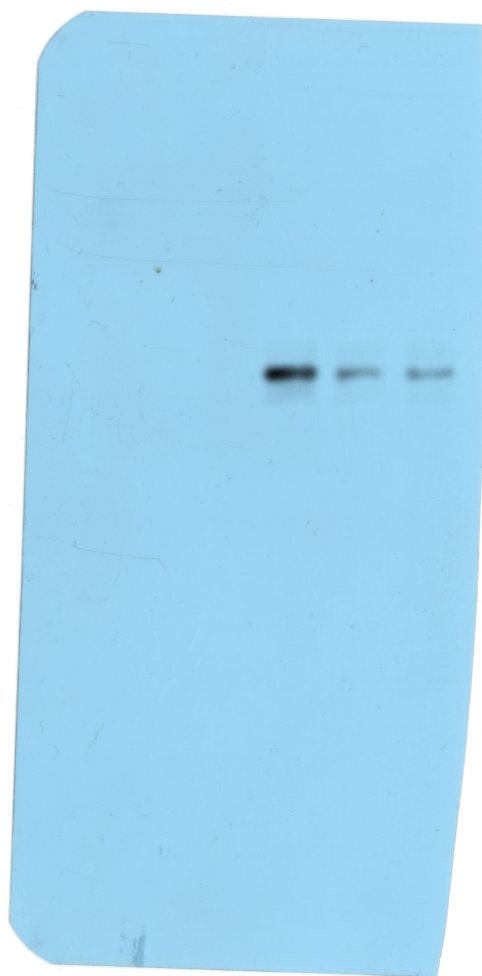

Supplement: Supplementary file 4 — Supplementary Information 4. [file 41598_2024_59676_MOESM4_ESM.pdf]

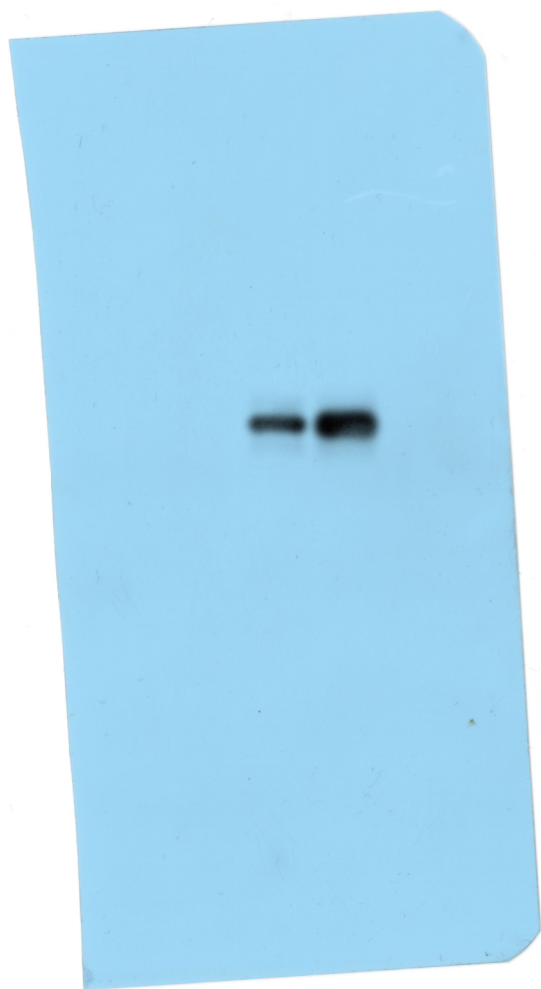

Supplement: Supplementary file 5 — Supplementary Information 5. [file 41598_2024_59676_MOESM5_ESM.pdf]

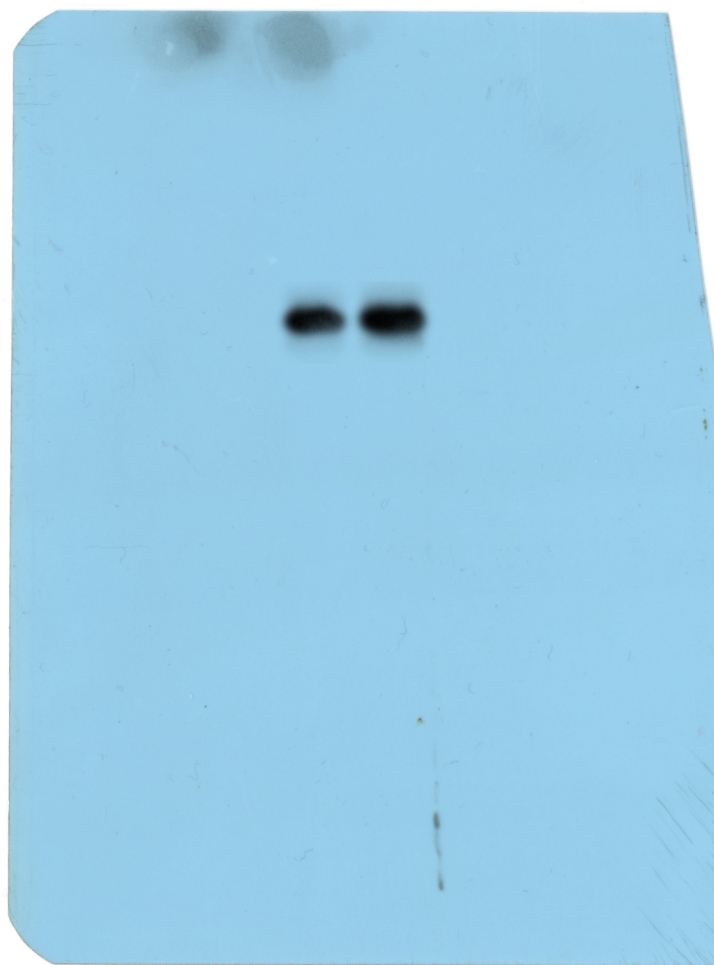

Supplement: Supplementary file 6 — Supplementary Information 6. [file 41598_2024_59676_MOESM6_ESM.pdf]

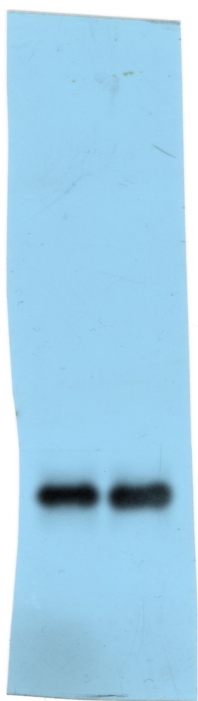

Supplement: Supplementary file 7 — Supplementary Information 7. [file 41598_2024_59676_MOESM7_ESM.pdf]

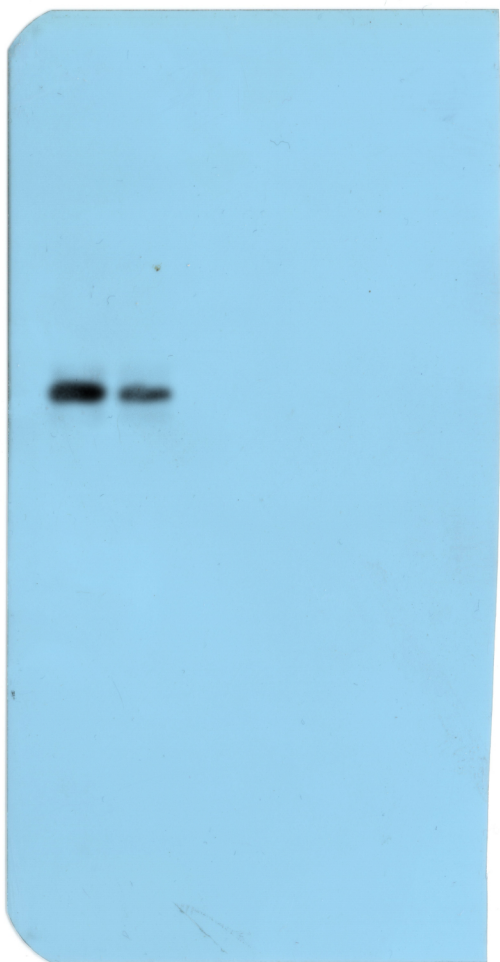

Supplement: Supplementary file 8 — Supplementary Information 8. [file 41598_2024_59676_MOESM8_ESM.pdf]

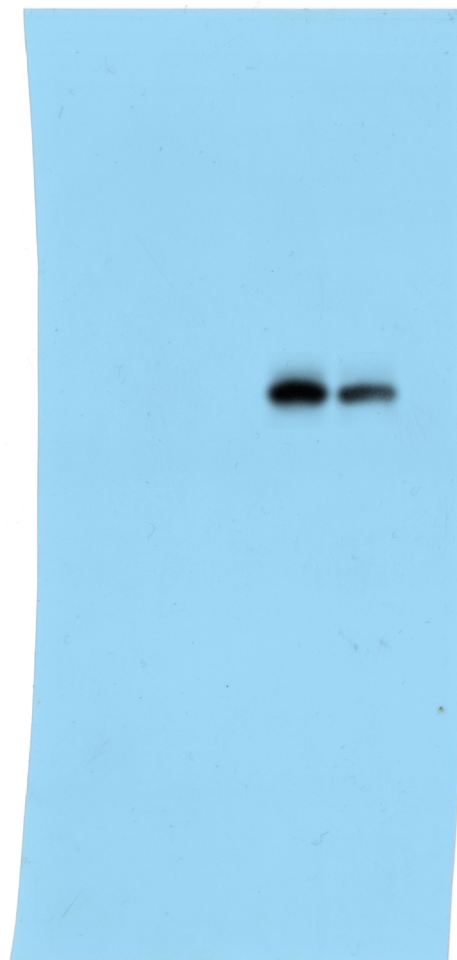

Supplement: Supplementary file 9 — Supplementary Information 9. [file 41598_2024_59676_MOESM9_ESM.pdf]

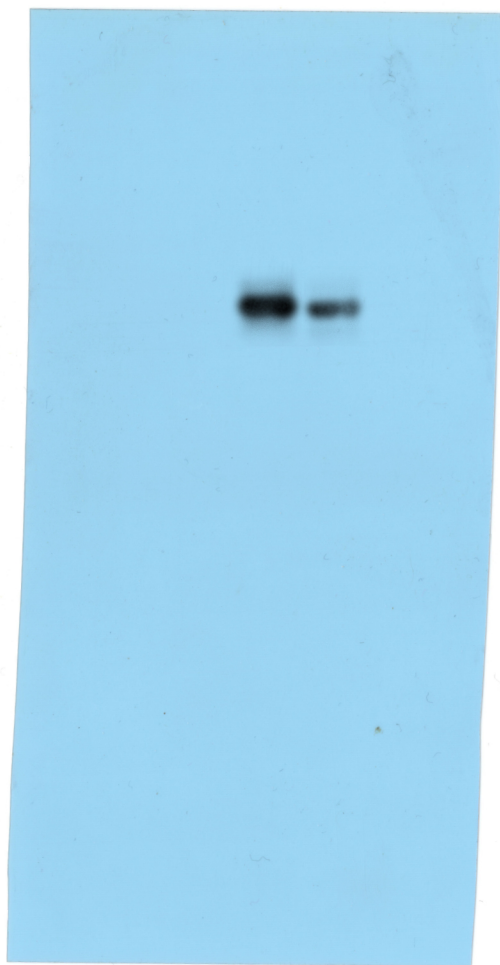

Supplement: Supplementary file 10 — Supplementary Information 10. [file 41598_2024_59676_MOESM10_ESM.pdf]

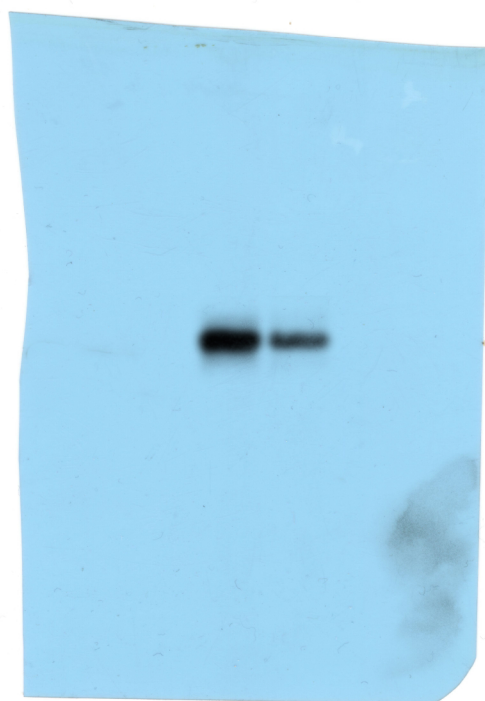

Supplement: Supplementary file 11 — Supplementary Information 11. [file 41598_2024_59676_MOESM11_ESM.pdf]
